# Metachronous Occurrence of Triple Malignancies of Kidneys, Prostate, and Breast. A Case Report and Review of The Literature

**DOI:** 10.1155/2013/194620

**Published:** 2013-03-06

**Authors:** Mohammad Ali Zargar-Shoshtari, Hossein Saffari, Mohammad Kazem Moslemi

**Affiliations:** ^1^Hasheminejad Kidney Center (HKC), Tehran University of Medical Sciences, Tehran, Iran; ^2^Department of Urology, School of Medicine, Ardabil University of Medical Sciences, Ardabil, Iran; ^3^Department of Urology, School of Medicine, Kamkar Hospital, Qom University of Medical Sciences, Qom, Iran

## Abstract

Multiple consecutive cancers involving different organs in a male individual are presented. *Case Presentation*. Herein, we present a rare case of primary right renal cell carcinoma (RCC), in which two different organ malignancies of prostate and breast were occurred consecutively. After proper treatment of each organ tumor, the patient experienced metachronous occurrence of its final tumor in his remained left kidney as left side RCC. *Discussions*. Multiple primary cancers are defined as occurrence of two or more malignancies, synchronous or metachronous, in different organs without any relation to each other. For primary and secondary tumors of the male genitourinary system, the most common occurrence was tumors of bladder and prostate followed by tumors of the kidney. Our case was a rare presentation of consecutive occurrence of multiple organ tumors: right side RCC, adenocarcinoma of prostate, and ductal cell carcinoma of the left breast, followed by left side RCC. *Conclusions*. In any case of primary malignancy of any organ, not only primary tumor recurrence but also tumoral growth of other nonrelated organs should be evaluated, especially in high risk patients or patients with positive familial history.

## 1. Case Presentation

**Figure 1:**
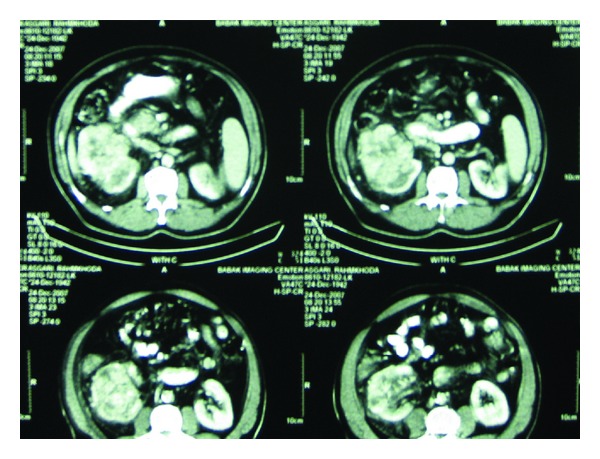
Contrast enhanced abdominal CT scan revealing the first tumor of the patient, right RCC.

The patient with known case of right RCC since 4 years ago was a 71-year-old Iranian who was nonsmoker, with medium built body (Figure 1). He been underwent right radical nephrectomy with preservation of the ipsilateral adrenal gland. Pathologic evaluation revealed an 8 × 6 cm mass of renal cell carcinoma, clear cell type, nuclear Fuhrman grade 2, with involvement of renal vein and perinephric fat (p T3b). After one year and as a routine followup, a serum prostate specific antigen (PSA) level of 7.9 ng/mL was detected. In the digital rectal examination, a 0.5 cm nodule was detected in the right lobe of the prostate gland, and finally, a trans-rectal ultrasound (TRUS) guided biopsy of the prostate was performed for him. Pathology report was in favor of adenocarcinoma of the prostate, Gleason score (3 + 4). The patient underwent retropubic radical prostatectomy RP. Adenocarcinoma of the prostate, Gleason score (3 + 4), with the focal surgical margin involvement without pelvic lymph node invasion was reported. The patient refused external beam radiotherapy (EBR) of the pelvic cavity. Then, hormonal therapy with monthly injection of LHRH agonist (Dipherlin; 3.75 mg; i.m) was initiated as adjuvant therapy. The PSA decreased to 0.1 ng/mL 3 months after RP. After one year, he mentioned a mass in his left breast. He underwent left radical mastectomy. Pathology report was in favor of poorly differentiated invasive ductal cell carcinoma.

**Figure 2:**
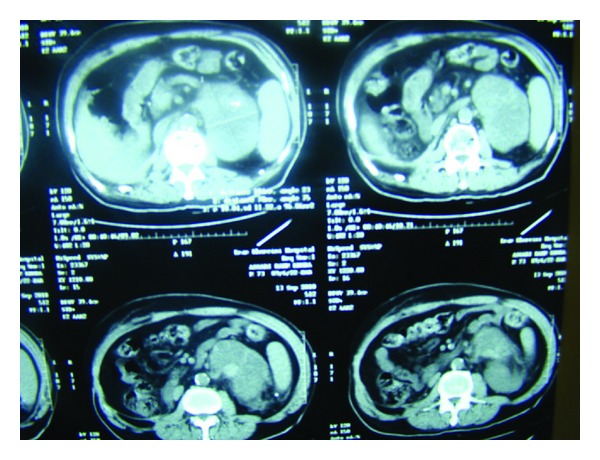
Contrast enhanced abdominal CT scan, revealing the left renal mass.

One year later and during followup for previous cancers, 11 cm mass was detected at the upper pole of the left kidney (Figure 2).

At the same time, all other studies such as liver function tests, bone scan, and chest CT scan were normal, without any sign of metastasis. Finally, the patient underwent left partial nephrectomy. Renal cell carcinoma, clear cell type, was reported too. The routine postoperative follow up studies were negative. One year later, the patient died due to cardiac arrest. 

## 2. Discussion

Multiple primary cancers are defined as occurrence of two or more malignancies, synchronous or metachronous, in different organs without any relation to each other [1]. Although secondary malignancies are rare, recently, they are increasingly reported in the literature [2]. The rate of multiple primary malignancies was reported as 4.5%–11.9% [3], 16.1% [4], and as high as 26.9% [5]. Beisland et al. [4] in a study of over 1425 patients with RCC found that 16% had one tumor, 1.6% had two tumors, and 0.2% had three other primary malignancies. Generally, 46.7% of tumors occurred as metachronous tumors. The most common second malignancy was the cancer of prostate. Eight cases of breast cancer as a second tumor were reported all in females. No male breast cancer was reported. A cumulative risk of developing second primary malignancy in males with RCC was found as high as 26.6% [4]. Beisland et al. concluded that patients with RCC have significantly higher risk of developing other subsequent primary malignancies. For the treatment of these lesions, the most aggressive tumor should be treated appropriately considering the age and general condition of the patient. After improving the overall general condition of the patient, other malignancies should be managed [6]. Using other diagnostic methods like ultrasonography, computed tomography (CT) scanning, and magnetic resonance imaging (MRI) for the primary evaluation of patients and follow-up of cases with undiagnosed and/or diagnosed malignancies helped us to better detect secondary cancers [7]. In addition and because of focusing on the involved organ system more precisely by the specialist, it is expected that urologist could detect prostate cancer and metachronous contralateral RCC with an increased frequency [8, 9]. However, the detection of other malignancies outside the organ system may be facilitated at the follow-up period.

In a study by Wegner [10], the occurrence of a second primary neoplasm was found in 144 cases (3.3%); over 4353 patients who were treated for urologic cancers over a 19 years period. He did not find any overall increased risk of secondary malignancies. However, in certain malignant associations, the risk was higher, with a factor of two, such as bladder and prostate and kidney and prostate [10]. 

Koyama et al. [11] studied 104 patients with multiple primary tumors and found an incidence of multiple tumor occurrences of 9% in patients with primary urologic tumors. He found a predominant occurrence of prostate and bladder cancers in urologic cancers and stomach cancer in nonurologic cancers. In his study, in seventy-six percent of cases, the second tumor was found within 5 years of primary tumor diagnosis. However, he concluded a strict follow-up in the first 5 years of primary tumor diagnosis.

Oka et al. [12] in a retrospective study of over 300 cases with RCC found an incidence of metachronous occurrence of RCC and prostate cancer in 6 cases (2%) with a mean follow-up period of 38 months.

Some case reports of synchronous or metachronous triple primary carcinomas were reported in the literature, such as triple synchronous involvement of the kidney, bladder and prostate cancers [13], or metachronous triple involvement of the bladder prostate, and renal cancers [13]. However, no case of triple primary cancer of kidney, prostate, and male breast cancer was reported in the literature. All the metachronous RCC with breast cancer was reported in female patients [14]. Arikan-Sengul et al. [14] studied seventeen female RCC cases associated with secondary gynecologic tumors. He found ten cases (60%) of RCC associated with breast carcinoma as the most common association. 

For primary and secondary tumors, the most common finding was tumors of bladder and prostate followed by tumors of the kidney and prostate [8]. The most common non-urologic secondary tumor after primary urologic tumor was colon cancer [4]. 

 Genetic, environmental, and dietary factors in addition to obesity have been suggested as etiologic factors for the development of prostate, kidney, breast, and colon cancers [3, 4]. For treatment of these lesions, the most aggressive tumor should be treated in appropriate with the age and general condition of the patient. After improving the overall patient general condition, other malignancies should be managed appropriately [5]. Increasing use of ultrasonography, CT scanning, and MRI for primary evaluation and follow-up of patients with undiagnosed and/or diagnosed malignancies helped us for early detection of secondary cancers [7]. In addition and because of focusing more precisely by specialist on the involved organ system, it is expected that urologist could detect prostate cancer and metachronous contralateral RCC with an increased frequency [4, 15]. However, the detection of other malignancies outside the organ system may be facilitated at the follow-up period.

## 3. Conclusion

Occurrence of multiple primary malignancies is still very rare. In any case with primary malignancy, urologic or nonurologic, investigation for other organ cancers is not warranted. Strict followup of the patient for primary tumor would help us for the early detection of emergence of new tumors.

Based on the best of our knowledge, this is the first case of triple metachronous cancers of bilateral kidneys, prostate, and breast reported in the literature.
